# Wheat Stem Rust Detection and Race Characterization in Tunisia

**DOI:** 10.3390/plants12030552

**Published:** 2023-01-25

**Authors:** Wided Abdedayem, Mehran Patpour, Marwa Laribi, Annemarie F. Justesen, Hajer Kouki, Moez Fakhfakh, Mogens S. Hovmøller, Amor H. Yahyaoui, Sonia Hamza, Sarrah Ben M’Barek

**Affiliations:** 1 National Agronomic Institute of Tunisia (INAT), 43 Avenue Charles Nicolle, Tunis 1002, Tunisia; 2 CRP Wheat Septoria Precision Phenotyping Platform, Tunis 1082, Tunisia; 3 Department of Agroecology, Aarhus University, 4200 Slagelse, Denmark; 4 Comptoir Multiservices Agricoles, 82, Avenue Louis Brailles, Tunis 1002, Tunisia; 5 Borlaug Training Foundation, Colorado State University, Fort Collins, CO 80523-1170, USA; 6 Laboratory of ‘Appui à la Durabilité des Systèmes de Production Agricole Dans la Région du Nord-Ouest’, Higher School of Agriculture of Kef (ESAK), Regional Field Crops Research Center of Beja (CRRGC) BP 350, Beja 9000, Tunisia

**Keywords:** stem rust, wheat, Tunisia, re-emergence, clade prevalence

## Abstract

Climate changes over the past 25 years have led to conducive conditions for invasive and transboundary fungal disease occurrence, including the re-emergence of wheat stem rust disease, caused by *Puccinia graminis* f.sp. *tritici* (Pgt) in East Africa, Europe, and the Mediterranean basin. Since 2018, sporadic infections have been observed in Tunisia. In this study, we investigated Pgt occurrence at major Tunisian wheat growing areas. Pgt monitoring, assessment, and sampling from planted trap nurseries at five different locations over two years (2021 and 2022) revealed the predominance of three races, namely TTRTF (Clade III-B), TKKTF (Clade IV-F), and TKTTF (Clade IV-B). Clade III-B was the most prevalent in 2021 as it was detected at all locations, while in 2022 Pgt was only reported at Beja and Jendouba, with the prevalence of Clade IV-B. The low levels of disease incidence during these two years and Pgt population diversity suggest that this fungus most likely originated from exotic incursions and that climate factors could have caused disease establishment in Tunisia. Further evaluation under the artificial disease pressure of Tunisian wheat varieties and weather-based modeling for early disease detection in the Mediterranean area could be helpful in monitoring and predicting wheat stem rust emergence and epidemics.

## 1. Introduction

Climate changes play a major role in pathogens’ emergence and dispersal in new ecological environments [1]. Moreover, the deployment of a limited number of resistance genes to fungal diseases worldwide could facilitate the evolution and emergence of new virulent strains, leading to resistance breakdown [2,3]. Stem rust (SR), caused by *Puccinia graminis* (Pg)*,* infects several grass and cereal hosts [4,5]. The *Puccinia graminis* f.sp. *tritici* Eriks & E. Henn (Pgt), which infects wheat, represents the most destructive disease that under favorable conditions could cause complete crop loss [6,7,8]. Pgt wind dispersal for long distances presents an epidemic risk and poses a threat to global food security [9,10]. The surveillance and monitoring of stem rust occurrence and dispersal are of major concern to national and global communities in wheat-producing areas. Several studies investigated the Pgt spread over countries and predicted potential pathways by developing models based on climate-related factors that control the success of the stem rust spores emission, atmospheric transport, disposition, germination, infection, and survival [11,12,13,14].

Over the recent decades, numerous areas, including East Africa [15], Europe [16,17,18], and Canada [19], reported the re-emergence of Pgt. The 1999 reports on *Sr31* breakdown in Uganda [20] and subsequently in Kenya, Ethiopia, Sudan, Yemen, and South Africa [21] lead to the identification of Ug99 race (TTKSK). The Ug99 race belongs to a genetic group, Clade I, comprising up to 15 races virulent to *Sr* genes [22] that are widely used in breeding programs worldwide. 

In contrast to bread wheat (BW), durum wheat (DW) was found to be less susceptible to the Ug99 race group; mainly attributed to *Sr13* gene that is present in most durum wheat varieties [21,23]. However, compared to BW, only a few stem rust resistance genes were identified in durum wheat [24,25,26].

In Tunisia, wheat stem rust was prevalent in the 1960s and 1970s [27]. Since the 1980s, no SR infection was reported for over three decades until spring of 2018 [28]. Pgt was recovered from survey data collected from Jendouba and Beja governorates in the northwestern regions, which revealed the presence of TKKTF race that belongs to Clade IV-F and TTRTF race [29], known as the ‘Sicily race’ (Clade III-B), initially identified in Italy in 2016 [16,30]. This latter race was reported to be virulent on several durum wheat varieties that possess the resistance gene *Sr13b* [15,23]. In 2020, TKKTP race was also identified from samples collected near Bou Salem (Jendouba) in northwestern Tunisia [31]. However, to date there is no information about the infection distribution, incidence, severity, and race composition of stem rust in Tunisia. 

The objectives of this study were to: (i) evaluate the distribution and relative occurrence of Pgt on a panel of durum and bread wheat varieties in different environments, (ii) investigate the relation between the climatic-related factors that control the success of stem rust occurrence, and finally (iii) identify and characterize the Pgt races that occurred in Tunisia since the re-emergence of the disease in 2018 [28].

## 2. Results

### 2.1. Stem Rust Disease Occurence in Tunisia

Surveillance and disease monitoring of trap nurseries at the different locations during two successive cropping seasons (2020–2021 and 2021–2022) revealed the presence of stem rust disease at five sites, including the experimental stations of Kodia (Jendouba) and Beja (Figure S1), as well as the farmer fields in Zaghouan, Mateur, and Bizerte in the 2020–2021 cropping season. In the 2021–2022 cropping season, Pgt was detected at low infection level only at Beja and Kodia experimental stations on the late sown trials (Figure S1). Because of the low incidence of stem rust in 2022, only the 2021 Pgt scoring data were considered for statistical analysis. Results showed that disease severity varied between the different testing areas (Table S3). Statistical analysis revealed no significant variance of Pgt infection between different accessions (*p* = 0.3775) but significant variance between sites (*p* ≤ 0.001) and the interaction sites x accessions (*p* = 0.0274) were noted (Table 1).

In Bizerte, stem rust disease was not detected on the accessions included in the trap nursery, despite the detection of the disease at two different farmer fields on late sown and untreated plants. Since the experimental trials were conducted under natural infection, the reported infection responses at the five tested sites presumably depends on the presence of Pgt spores in the air. The total absence of stem rust on the universal susceptible genotype ‘Morocco’, as well as on ‘McNair-701’ in Beja (Table S3) indicates that the disease was not well established and the reported resistance infection responses (R) could be due to disease escape. 

Recorded infection type on stem rust differential lines was the highest at Kodia-Jendouba (more than 50% of differential lines displayed an MS or S infection responses), followed by Zaghouan and Mateur that showed similar infection level (5% of the differential lines showed a susceptible infection response) (Table S3). Conversely, the distribution of the differential lines according to infection response categories showed a lower level of disease occurrence at Beja (Table S3). At Kodia experimental station, the highest infection at adult growth stage was recorded on the differential lines ‘Festiguay W2706 PI330957′ and ‘LCSR24Ag’, carrying the resistance genes *Sr30* and *Sr24*, displaying disease severity and infection response of 60 S and 50 S, respectively, while at the other sites they showed a lower infection level (Table S3). Moreover, the differential line ’SR31/6*LMPG’ carrying the resistance gene *Sr31*, which is associated with the detection of Ug99 race (TTKSK), displayed resistance (R-MR) at all sites. The 43 accessions tested in this study in addition to the SR differential lines mostly displayed low infection levels (showing an R and MR infection types). Whereas 16% of accessions showed a variation in their reaction to stem rust infection between sites, about 30% of the tested accessions had a stable reaction (R or MR) across the different sites, such as the triticale variety ‘Bicentenario’, displayed an immune reaction to Pgt at the four sites (Table S3).

### 2.2. Genotyping and Race Phenotyping

**Figure 1:**
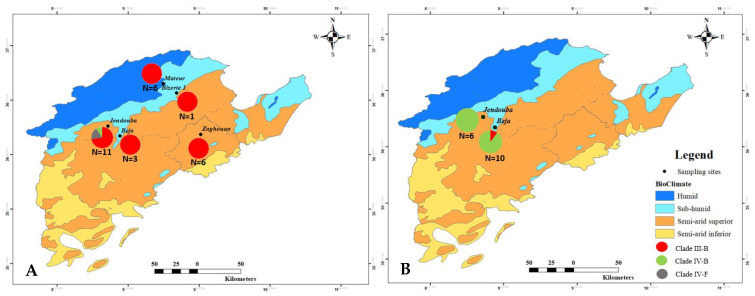
Pgt sampling sites (black dots) and identified clades frequencies based on number of samples (N, mentioned below pies) per site from farmer fields and trap nurseries in the northwestern region of Tunisia on 2021 (**A**) and 2022 (**B**) cropping seasons.

Forty-three stem rust samples collected from different locations in the northwestern region of Tunisia during two successive cropping seasons were genotyped using SSR markers. In 2021, 27 wheat stem rust samples have been collected from different sites; Beja, Bizerte (Farmer field), Zaghouan, Mateur, and Kodia-Jendouba. As a result, three clades were recorded, with the dominance of Clade III-B (Figure 1A, Table S2). However, in the 2022 cropping season, from a total of 16 samples collected from Beja and Kodia-Jendouba, 15 samples belonged to Clade IV-B and only 1 sample belonged to Clade III-B (Figure 1B, Table S2). Reported clade frequencies at different sampling locations during the two cropping seasons are illustrated in Figure 1.

**Figure 2:**
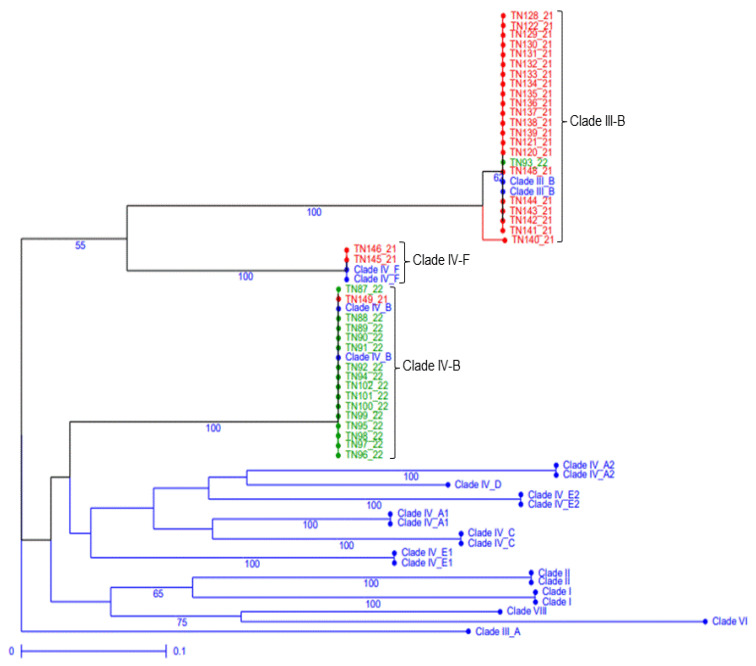
Consensus tree of 40 Pgt (3 isolates were not considered due to incomplete SSR data) isolates collected from Tunisia in 2021 and in 2022, presented in red and green colors, respectively, and 24 reference representative isolates of different Pgt clades [18], presented by their corresponding clade in blue color, based on genotyping data using 17 Pgt SSR markers [18]. Bootstrap values supporting the cluster at higher than 50% after 1000 iterations are shown above the internodes.

Overall, results showed that 58.1% of the samples belonged to Clade III-B corresponding to the ‘Sicily race’, race TTRTF, while 37.2% of the samples belonged to Clade IV-B, race TKTTF, and only 4.7% represented Clade IV-F with race TKKTF (Figure 2, Table S2). Race phenotyping of the twenty SR North American differentials set confirmed the SSR genotyping results (Table S2). TTRTF race was recovered from four samples that have been collected from Bizerte, Zaghouan, and Kodia-Jendouba during the 2021 cropping season and from one sample collected in 2022 at Beja. The remaining five phenotyped isolates of 2022 samples showed race TKTTF, which belonged to clade IV-B in both areas, Beja and Kodia-Jendouba (Table S2). 

### 2.3. Climatic Factors Effect on Pgt Occurrence in Tunisia

**Figure 3:**
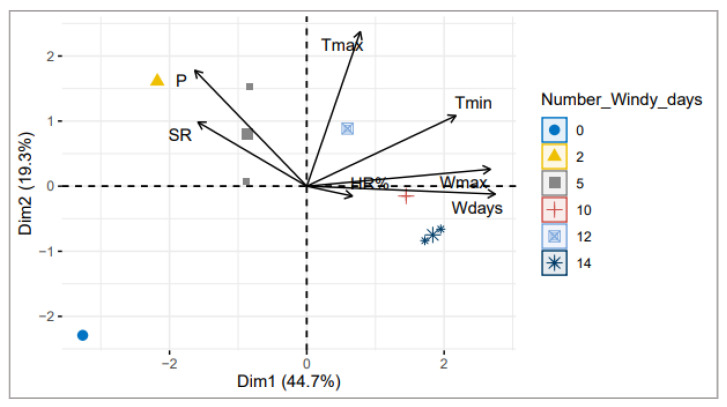
Distribution of SR trap nurseries clustered based on the number of windy days (Wdays) on two dimensions principal component analysis (PCA) plot of disease occurrence (SR), P (precipitation, mm), Tmax (average maximum temperature, °C), Tmin (average of minimum temperature, °C), HR% (average of relative humidity, %), Wmax (average of maximum wind speed), and Wdays (number of windy days) during the three spring months (March, April, and May) of both 2021 and 2022 cropping seasons.

To further investigate the climatic factors variation effect on wheat stem rust disease occurrence in the fields, we applied the principal component analysis (PCA). Analysis showed two PCA dimensions explaining 64% of variance (Figure 3). Wmax, Wdays, and Tmin contributed greatly to the first dimension of PCA by 67.59%. The higher contribution to the latter component was measured for Wdays and Wmax with the highest correlation coefficient (r = 0.95 and r = 0.93, respectively, *p* < 0.01). The second dimension contributed by 79.63% by Tmax and P with a significant correlation coefficient of r = 0.82. SR incidence negatively correlated to Wdays (r = −0.66 with *p* < 0.05) and was lower to Wmax (r = −0.31) (Figure 3). 

Results showed that stem rust disease occurrence in the Tunisian environment is mostly affected by the wind factor, suggesting that a high number of windy days during the three spring months, where wind speed exceeds 60 km/h, decreases SR establishment and development on the host plant. The highest number of Wdays was recorded in Zaghouan and Mateur (14 days during the months of March, April, and May) during the 2021–2022 cropping season and where the disease was not detected. 

## 3. Discussion

### 3.1. Pgt Occurrence in the Tunisian Environment

In our study, the detection of wheat stem rust disease in Tunisia at multiple locations illustrates that the disease can occur in wheat-growing areas under conducive conditions and emphasizes the need to initiate local monitoring for this re-emergent threat to wheat production across the country and North Africa. This can be performed using sets of differential lines for Pgt trapping and disease surveillance, as conducted in this study. Trap plots to assess rust diseases presence/absence have been shown to provide useful information about virulence/avirulence of local Pgt population for specific resistance genes in order to support the global cereal rust monitoring system (GCRMS) [32]. However, the effectiveness of trap plots under field/natural infection could be affected by local environmental conditions and Pgt inoculum concentration in the air, as observed in this study. As a matter of fact, the difference in stem rust disease incidence and occurrence was clearly visible and varied from place to place, depending on agroecological divergence, variety grown, date of sowing, and from year to year. In general, infection responses in the tested wheat accessions mostly showed a low infection level compared to the differential lines that showed a higher variability in disease level. However, reported resistance on the tested accessions could be due to disease escape, particularly that the susceptible variety ‘Morocco’ was not infected at all sites. Hence, for more accurate evaluation of stem rust disease resistance/tolerance of the wheat accessions used in this study, further investigation should be conducted using artificial inoculation under controlled conditions, as well as in the field.

Moreover, climatic factors play an important role in disease spread and emergence. For rust diseases, three main potential effects of climate change have been highlighted: the fast evolution of new pathotypes and shifting virulence under favorable conditions, the reduced effectiveness of rust resistance, and the pathogen spread over large areas [33,34,35]; the latter effect being mainly driven by long-range atmospheric transport. In this context, several weather-based infection models for wheat stem rust, based on temperature, relative humidity, precipitation, and wind/ air circulation, have been developed to predict the potential disease emergence in the future [13,36]. Prank et al. [12] suggested that future simulations of drier and more turbulent conditions will allow a larger fraction of the urediniospores emission from infection source fields to the atmosphere. In the present study, the PCA analysis showed a significant negative correlation between SR occurrence and both maximum average of wind speed and the number of days, where the wind speed exceeds 60 km/h during the three spring months, March, April, and May. Wind circulation and air turbulence not only maintain the spores in movement, thereby decreasing the amount of deposited urediniospores at high wind speed, but also affect the microclimate on the wheat leaf or stem surfaces by preventing disease development on the surface as the leaf becomes dry [12]. The wind factor during the spring period most likely plays a key role on rust dispersal and disposition on the vegetative canopy in Tunisia. In addition, the large-scale atmospheric dispersal simulation model developed by Prank et al. [12] proposed that travelled distances in one day can reach a few thousand kilometers with strong winds, allowing frequent exchange of viable spores between Europe and North Africa. 

The SR infection level variance between regions and host genotypes may be a result of a combined effect of climatic factors, potential aerial dispersal, Pgt urediniospores viability and concentration in the air, host resistance, and also agronomic practices (planting date). This latter factor could explain why Pgt was not detected in the trap nursery planted in Bizerte in the 2021 cropping season but detected on late planted durum material of the farmer’s fields in the same region. Similarly, the widely cultivated durum wheat variety ‘Karim’ that is highly appreciated by local farmers due to its high yield and wide adaptation to the local environment [37] showed a resistance infection response when planted in November (regular planting time) and an MS-S infection response when planted late in the season. As a matter of fact, in the 2022 cropping season, all samples were collected from the late planted material. Several studies reported the effect of the planting date in association with maturity on rust disease’s progression and severity [38]. For instance, Naseri and Sabeti [39] suggested that the early sowing of early-maturing genotypes could reduce stem rust intensity as effectively as genotypic resistance while late-sowing increased stem rust epidemics under favorable conditions. Similarly, Southern and Wilcoxson [40] reported that the planting date had an impact on the slow rusting in resistant wheat varieties against Pgt. As a matter of fact, Tunisian farmers are unintentionally eliminating the risk of disease occurrence as, in general, wheat plants are in an advanced growth stage (maturity) at the time of Pgt spores’ disposition (end of May–beginning of June).

### 3.2. Race Dominance in Tunisia and Possible Infection Source

In Tunisia, stem rust disease was prevalent in the 1970s [27,41]. Three races, namely 11, 14, and 24 [27], were identified based on 12 differential lines set defined by Stakman in 1922 [39]. Races 14 and 24 were detected in the regions of Fahs (Zaghouan) and Beja, respectively, while race 11 was found at both Tunis and Mateur governorates. The three races were avirulent to *Sr14* and to *Sr6* with an exception for biotype 11 that originated from Tunis. On the other hand, only races 11 and 12 were virulent to *Sr9a* [27]. Since 1988, Roelf and Martens [42] established an international system for Pgt race nomenclature. Even so, the conversion into the new letter code designation was not reliable, and it was not possible to study the evolution of these races in Tunisia or to compare their lineage of to the newly identified ones. Additionally, races, such as 11 and 14, which originated from North African and European countries, had more than one code designation, thereby representing different biotypes [42]. Several decades later, its first detection of SR was reported in the northwestern regions of Tunisia, particularly in Beja and Jendouba [29,31]. 

Molecular analysis of 43 samples collected in this study from different sites in the northwestern region of Tunisia, and during two-cropping seasons (2021 and 2022), revealed the presence of three clades: clade III-B, IV-B, and IV-F. In 2021, Clade III-B, referred to the ‘Sicily’ race, was the most dominant (TTRTF). The TTRTF race was firstly reported in Tunisia in 2019 [29] and later in 2021 and 2022 (this study) at different frequencies between the seasons. As its name indicates, the ‘Sicily’ race was detected and identified for the first time in durum wheat fields in Sicily (Italy) and caused an epidemic during the 2016–2017 cropping season [16,30]. However, it has been present in Georgia since 2014 [43] and in Azerbaijan in 2015 [44]. It has also been detected in other regions, including Eretria (in 2016) [45], Egypt (in 2018) [46], Ethiopia (in 2018) [47], Hungary (in 2019) [43], and Iran (in 2019) [48]. The ‘Sicily’ race is one of the most virulent races on durum wheat, in addition to race JRCQC [49]. It was shown to be virulent to *Sr5, Sr21, Sr9e, Sr7b, Sr11, Sr6, Sr8a, Sr9g, Sr36, Sr9b, Sr17, Sr9a, Sr9d, Sr10, SrTmp, Sr38,* and *SrMcN* but avirulent to *Sr30, Sr24,* and *Sr31*. Additionally, it has a wide virulence spectrum that includes 23 *Sr genes*, [45] namely *Sr13b* and *Sr50*, which were identified in durum wheat and rye, respectively. The two latter genes were found to be effective against the Ug99 group [21]. Yet, *Sr13a* is still effective against TRTTF and JRCQC races to date [23,43]. 

In addition, two samples collected from Kodia-Jendouba experimental stations in the 2021 cropping season belonged to clade IV-F, known as the TKKTF race. Previously, the TKKTF race was genetically classified into Clade IV-E [43]. This race recently reported in Tunisia in 2019 is characterized by its avirulence to *Sr11*, *Sr36*, *Sr24,* and *Sr31* [27]. TKKTF is geographically widely distributed across Europe, West Asia, and Africa, including Morocco [18,43,50].

In this study, we detected the TKTTF race that belonged to clade IV-B for the first time in 2021, which was the most prevalent clade in 2022. This race, also known as the ‘Digalu’ race, differs only by an additional virulence to *Sr36* compared to race TKKTF. The TKTTF race was firstly detected in Ethiopia in 2012 and reached an epidemic level in 2013 by overcoming the *SrTmp* resistance gene on the popular cultivated variety ‘Digalu’ in Ethiopia [6]. Clade IV-B was previously reported in numerous Mediterranean countries, such as Italy, Morocco, and Spain [18,29,50].

Overall, the genotyping and phenotyping analyses confirmed the dominance of a restricted number of races or clades (Clade III-B, IV-B, and IV-F) in Tunisia that could be detected in conducive conditions with a change in frequency over time (compared to findings in 2019 [29], 2021, and 2022 (current study)). 

Excluding the possibility of Pgt sexual recombination in Tunisia for which no SR alternate hosts (*Berberis* spp.) have been reported so far [51] and the low diversity level of stem rust infections mainly driven by clonal reproduction, stem rust was more likely to have been derived from an influx of windborne Pgt urediniospores. Interestingly, these three clades were all detected in the Mediterranean area since 2018 with different frequencies over time and placed prior to their detection in Tunisia [18,29,50,52]. Furthermore, despite the absence of studies on race composition in the neighboring country of Algeria, where the alternate host is present [53,54], the disease was detected sporadically in 2018 [55]. Hence, further race analysis may give more insights into the current situation of stem rust and the potential role of the barberry plants in the occurrence of SR in Algeria. 

## 4. Materials and Methods

### 4.1. Plant Material and Experimental Design

During two successive cropping seasons (2020–2021 and 2021–2022), trap nurseries consisting of 78 accessions were planted in a complete randomized design at five different sites in the northern region of Tunisia; two sites were at the experimental stations of the CRP-Wheat Septoria precision phenotyping platform, namely Beja and Kodia (Bou Salem-Jendouba), while the other three sites were located at farmer fields in the regions of Bizerte, Zaghouan, and Mateur. In 2022, trap nurseries were also planted at late sowing dates at the two experimental stations (Beja and Kodia) (Table 2). Both trap nurseries comprised the North American differential set of stem rust (#20), Tunisian durum wheat landraces (#14), commercial bread (#16), and durum (#27) wheat varieties, in addition to triticale (#1) and the susceptible genotype ‘Morocco’; all accessions and additional information over two year’s experiments are listed in Table S1. 

### 4.2. Disease Assessment and Sampling

Stem rust disease assessment was conducted under natural infection conditions during the month of May, from May 7 to May 21, corresponding to adult growth stages GS 83–90 [56]. Disease reactions based on severity were classified according to the modified Cobb scale [57] and infections categories recorded as resistant (R), moderately resistant (MR), moderately susceptible (MS), susceptible (S), and mixed infections (X) when two or more infection categories were recorded on the same stem. In addition to the infection response, the percentage of stem infected area was also recorded. Following the Borlaug Global Rust Initiative (BGRI) method described by Park et al. [58], a total of 43 samples were collected during the two cropping seasons from trap nurseries and also from accessions present at both experimental stations and farmer fields. As the disease is sporadically detected in Tunisia, maximum samples were collected from different locations; one sample per genotype in order to keep infected materials for disease assessment under field conditions. In the 2021 cropping season, we were able to collect infected stems from Jendouba, Zaghouan, Beja, and Mateur trap nurseries. However, in Bizerte, stem rust disease was detected sporadically on late sowing wheat material at farmer field, and no infection was observed on the trap nursery (Table S2). With lower frequency, stem rust was only detected at Beja and Jendouba during 2022 season, and only 16 samples of stem rust were collected. 

After collection, green samples were dried for 48 h at room temperature and sent to the Global Rust Reference Center (GRCC) in Denmark for clade identification.

### 4.3. Genotyping and Race Phenotyping

Forty-three stem rust samples (infected stems and/or leaves) that have been collected from different sites in the northwestern region of Tunisia (Table S2) were analyzed at the Global Rust Reference Center (GRRC) in Denmark. Following the protocol described by Patpour et al. [18], Pgt genomic DNA was extracted from infected stems using the Sbeadex^®^ Mini Plant Kit (LGC Genomics, Germany) and subsequently genotyped using seventeen Pgt simple sequence repeat (SSR) markers previously applied by Stoxen, Jin, and Zhong [59,60,61]. The amplicons were visualized in GeneMarker^®^ (V2.6.3); allele sizes were manually scored, as described in Patpour et al. [18], and genotyping data of well characterized 24 isolates representing the different clades were used as reference [18], in addition to the Tunisian isolates.

Among 43 samples, only 20 were recovered as the remaining samples, particularly those of 2021, were too dry. Finally, 10 were selected for race phenotyping (4 isolates from 2021 and 6 isolates from 2022, representing the two detected clades (Table S2). 

Samples were incubated in misted Petri dishes for 1 to 2 days at 18 °C in the light to promote urediniospores formation and recovering. After spore increase in selected samples for race phenotyping on the universally stem-rust-susceptible wheat plants ‘Morocco’, the twenty North American differential lines set of stem rust [62] were inoculated and scored based on the 0–4 scoring scale [63] at 16 days post-inoculation. Information about number of samples per year and per site is summarized in Table S2. 

### 4.4. Statistical Analysis

The effect of genotype and sites on stem rust disease infection responses were investigated using two way analysis of variance (ANOVA) with JMP (JMP 16.0; SAS, Inc., Cary, NC, USA); the infection response for each accession was converted into a constant value (where R, MR, M, MS, and S infection types are assigned to the constant values 0.2, 0.4, 0.6, 0.8, and 1, respectively) and multiplied by the terminal disease severity to derive a coefficient of infection [41]. Principal component analysis (PCA) was performed using the R package ‘MASS’ (R software version 4.1.2; Vienna, Austria) [64]. The coefficient of correlation between variables (SR infection level, average of maximum and minimum temperature (°C) (T_max_ and T_min_, respectively), precipitation (mm) (P), relative humidity (%) (RH%), maximum wind speed (Km/h) (Wsmax), and number of windy days (Wind speed > 60 km/h) (Wdays)) reported during the three spring months (March, April, and May) was determined with ‘cor.test’ function from the R package ‘stats’ [65]. The climatic factors data were obtained from the Tunisian National Institute of Meteorology (https://www.meteo.tn/en, accessed on 15 September 2022). Samples genotyping data analysis was visualized using the unweighted neighbor joining (uNJ) tree (with 1000 bootstraps) generated using the SSR data of Tunisian *Pgt* samples, in addition to 24 reference isolates, representing the different genetic groups (clades) used by Patpour et al. [18], based on the dissimilarity matrix between the individuals, as implemented in the DARwin 6 software [66].

## 5. Conclusions

This study confirmed the re-emergence of wheat stem rust disease in Tunisia and indicated the prevalence of two clades, clade III-B and clade IV-B in the 2021 and 2022 cropping seasons, respectively. Clade IV-F was also found but at a low frequency. The ability of rust spores to spread over long distances and the conductive climatic conditions for disease infection and development, as well as Tunisia’s proximity to countries where the disease has been reported, such as Spain, France, Italy, and Morocco, constitute a risk for the disease spread in new wheat-growing areas, even though the alternate host plant was not reported in the country. Further surveys and virulence tests should be conducted under artificial inoculation for more accurate disease evaluation of Tunisian cultivated wheat varieties and landraces to Pgt races that may harbor novel SR-resistant genes and be further exploited in breeding programs.

The surveillance of wheat stem rust occurrence or spread, including incidence assessments and virulence characterization via either trap plots or race (pathotype) surveys, has provided fundamental information. Future weather-based modeling for early disease detection in the Mediterranean basin could be helpful in monitoring and predicting wheat stem rust emergence and epidemics. 

## Figures and Tables

**Table 1:** ANOVA test of the reported stem rust infection response in the trap nurseries’ accessions under natural disease pressure in field conditions at the five different tested sites in Tunisia during the 2021 cropping season.

Source of Variation	Df	Square Sum	F Value	*p*-Value
Sites	4	2319.405	16.002	<0.0001 ***
Accessions	62	2864.556	1.057	0.3775
Sites × Accessions	205	6917.802	843.634	0.0274 *

Significance codes: ‘*’ 0.05, ‘***’ 0.001.

**Table 2:** Sowing areas and dates of the stem rust trap nurseries during the two successive cropping seasons 2020–2021 and 2021–2022 in Tunisia.

		Sowing Areas				
		Beja	Jendouba	Zaghouan	Mateur	Bizerte
		Septoria Platform Experimental Station	Kodia-Septoria Platform Experimental Station	Farmer Field	Farmer Field	Farmer Field
		2020–2021 cropping season				
Sowing date		4-Nov	6-Nov	13-Nov	15-Nov	16-Nov
N° of replicates/site		1	1	1	1	1
		2021–2022 cropping season				
Sowing date	Early	26-Nov	18-Nov	23-Nov	20-Nov	-
	Late	24-Dec	23-Dec	-	-	-
N° of replicates/site		2	2	2	2	-

## Data Availability

Not applicable.

## Supplementary Materials

The following supporting information can be downloaded at: https://www.mdpi.com/article/10.3390/plants12030552/s1, Figure S1: Stem rust disease symptoms detected in 2021 (A–C) and 2022 (D) cropping seasons at different locations in Tunisia within the trap plots; Table S1: List of planted genotypes on the trap nurseries during 2020–2021 and 2021–2022 cropping seasons; Table S2: Stem rust detected races and clades from Tunisian collected in 2021 and 2022 seasons; Table S3: Recorded SR infection types on the trap nurseries planted in 2020–2021 crop season in Jendouba, Beja, Zaghouan and Mateur.
