# Supplementary material for: Wheat Stem Rust Detection and Race Characterization in Tunisia
Source: Plants (Basel). 2023 Jan 25;12(3):552. doi: 10.3390/plants12030552 (PMC9919909; doi:10.3390/plants12030552)
Supplement: Supplementary file 1 [file plants-12-00552-s001.zip › plants-2059697-supplementary/supplementary-Tables&Figure/Supplementary-Figure-plants.pdf]

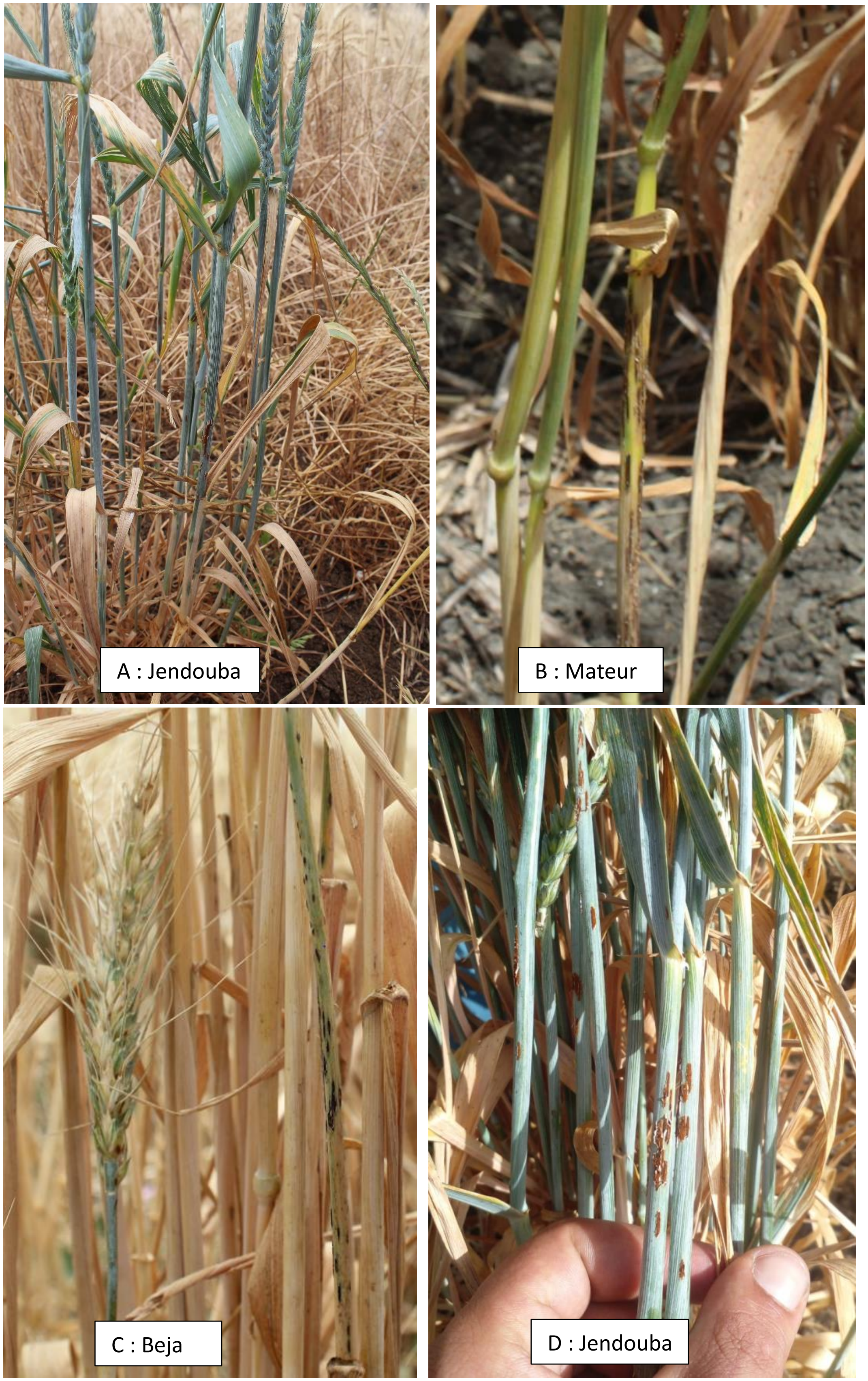


**Figure S1:** Stem rust disease symptoms detected in 2021 (A, B and C) and 2022 (D) cropping seasons at different locations in Tunisia within the trap plots.
